# A Computer Numerical Control Wire Electrical Discharge Machining Strategy for Fabricating Cobalt–Copper Bimetallic Oxide Maze-like Micro-Supercapacitors

**DOI:** 10.3390/mi17050516

**Published:** 2026-04-23

**Authors:** Ziliang Chen, Rui Xie, Chunlong Chen, Yiwei Zheng, Jianping Deng, Dawei Liu, Binbin Zheng, Wenxia Wang, Igor Zhitomirsky, Ri Chen

**Affiliations:** 1 School of Electromechanical Engineering, Guangdong Polytechnic Normal University, Guangzhou 510450, China; chenziliang@stu.gpnu.edu.cn (Z.C.); xierui@stu.gpnu.edu.cn (R.X.); chenchunlong@stu.gpnu.edu.cn (C.C.); zhengyiwei@stu.gpnu.edu.cn (Y.Z.); dengjianping@stu.gpnu.edu.cn (J.D.); binbinzheng@gpnu.edu.cn (B.Z.); 2 Biomedical and Pharmaceutical Sciences School, Guangdong University of Technology, Guangzhou 510006, China; fewwxia@gdut.edu.cn; 3 Materials Science and Engineering Department, McMaster University, Hamilton, ON L8S 4L8, Canada; zhitom@mcmaster.ca

**Keywords:** CoCuO_x_, micro-supercapacitor, binder-free, computer numerical control, wire electrical discharge machining, oxygen vacancy

## Abstract

Cobalt–copper bimetallic oxides (CoCuO_x_) show great potential for constructing high-performance micro-supercapacitors (MSCs) for micro-electronic applications. However, their poor conductivity and complex preparation procedures significantly hinder their broad applications. To address these challenges, oxygen-vacancy-modified CoCuO_x_-based binder-free electrodes were fabricated using a one-step computer numerical control wire electrical discharge machining (CNCWEDM) strategy. This approach enabled the fabrication of CoCuO_x_-based maze-like MSCs (CoCuMMSCs) with designable electrochemical performance, which could be simply controlled by their geometric shape and machining voltage. Subsequently, theoretical simulations were conducted for studying the effect of MSCs geometric shape on their capacitive behavior. Remarkably, the CoCuMMSCs fabricated by a machining voltage of 100 V achieved the maximum capacitance of 32.8 mF cm^−2^ at 0.15 mA cm^−2^. Furthermore, the CoCuMMSCs demonstrated outstanding performance at ultrahigh scan rates of up to 50,000 mV s^−1^, exceeding by more than two orders of magnitude the values previously reported in the literature. The obtained results proved that the development of the CNCWEDM technique facilitated manufacturing CoCuMMSCs devices with excellent performance by the comprehensive utilization of oxygen-vacancy incorporation, synergistic effect of cobalt and copper oxides, binder-free electrode design, proper device construction and controllable machining voltage. The advanced CNCWEDM strategy creates a new pathway for the high-efficiency fabrication of high-performance bimetallic-oxide-based micro-electronic devices, such as MSCs, intelligent micro-sensors and micro-batteries.

## 1. Introduction

With the growing demand for miniaturized, portable, implantable, and wearable electronic products, contemporary society urgently requires the development of customized, integrated microelectronic energy storage devices with enhanced capacity to power them [1,2,3,4,5]. At present, the majority of commercially available microdevices are powered by micro-batteries, which are distinguished by their ability to deliver high energy density. However, micro-batteries possess certain disadvantages, including a limited cycle life, a low power density, and adverse effects on the environment [6,7,8,9,10]. In comparison with micro-batteries, micro-supercapacitors (MSCs) have emerged as a pivotal energy storage solution in the development of contemporary micro-electronic products. This can be attributed to the merits of MSCs, such as high capacitance, high power density, superior cycling stability, good safety, easy maintenance, and environmental sustainability [11,12,13]. According to their charge storing mechanism, MSCs could be mainly classified into two groups, including electric-double-layer-capacitors (EDLCs) and pseudocapacitors (PCs). Carbon-nanotubes (CNTs), activated carbon, reduced-graphene-oxide (rGO), graphene-oxide (GO), carbon nanostructures, and other carbon-based materials belong to EDLCs, which exhibit excellent electrical conductivity, large surface area, and outstanding chemical stability [14,15,16]. However, these carbon-based materials offer very limited specific capacitance [17,18,19]. Compared with EDLCs, PCs demonstrate remarkably higher specific capacitance and specific energy density triggered by redox reactions. Transition metal oxides, conducting polymers and transition metal sulfides (TMSs) are promising candidates for PCs [20,21,22]. Conductive polymers, like polypyrrole (PPy), polyaniline (PANI), PEDOT:poly (4-styrene sulfonate) (PEDOT:PSS) and poly(3,4ethylenedioxythiophene) (PEDOT), are under investigation as promising electrode materials for PCs [23,24]. However, during charging and discharging, these conductive polymers undergo significant volume changes which result in their mechanical failures [25]. TMSs also demonstrate great potential for PCs application because of their high theoretical capacitances and high energy densities [26,27]. Unfortunately, metal sulfides are susceptible to several limitations, including volume expansion during cycling, slow reaction kinetics, and concurrent side reactions [28,29]. Bimetallic oxides (BMOs) have drawn considerable attention because of their superior energy density compared to carbon materials, enhanced stability over conductive polymers, reduced toxicity, environmental friendliness, and cost effectiveness [30,31,32]. A variety of TMOs, including FeCo_2_O_4_ [33], NiMn_2_O_4_ [34], ZnMn_2_O_4_ [35], Ni_x_Co_3−x_O_4_ [36], CoMn_2_O_4_ [37], Co_3_V_2_O_8_ [38], CuMoO_x_ [3,39], CuCo_2_O_4_ [40], CuCoO_2_ [41], and Cu_x_Co_3−x_O_4_ [42], have been extensively developed for PC electrode materials owing to their advantageous properties, such as considerable capacitance value, multiple valence states beneficial for fast redox reactions, and the synergistic effect of two different metal oxides with respect to their single components [43]. Among these BMO materials, CuCoO_x_ is considered a promising PC electrode candidate due to its high theoretical capacitance, low cost, and environmental friendless [44]. However, CuCoO_x_ has been limited by its low electrical conductivity and limited cycling stability when utilized as materials for PC electrodes [40,44].

To address these challenges, numerous studies have focused on developing CuCoO_x_ nanostructures with plentiful active sites for boosting the electronic/ionic transport efficiency [40]. For instance, Kadam et al. [45] used a chemical deposition technique with the assistance of silicone oil bath for synthesizing rose-like CuCo_2_O_4_ nanostructures on 3D nickel foam for PC application. Owing to the fast electronic transport efficiency achieved by the rose-like nanostructure of CuCo_2_O_4_ and the binder-free electrode configuration, the CuCo_2_O_4_ electrode demonstrated a superior capacitance value of 770 F g^−1^ (4 mA cm^−2^) in 2M KOH electrolyte. Ensafi et al. [46] also fabricated hollow-sphere nanostructured CuCo_2_O_4_ with high surface using a combined technique of solvothermal reaction, the hydrothermal method and the annealing method. The fabricated CuCo_2_O_4_ electrode showed outstanding capacitive performance because its unique nanostructure provided sufficient active sites for fast kinetics. Many investigations focused on the preparation of CuCoO_x_-based composite materials to enhance their electric conductivity [47,48,49]. For example, Azzou et al. [41] adopted a hydrothermal strategy to synthesize CuCoO_2_/activated-carbon composite materials for PCs application. Their electrochemical testing results verified that the introduction of activated carbon greatly enhanced the pseudo-capacitive behavior of CuCoO_2_. Bhagwan et al. [50] developed a co-precipitation method for anchoring CuCo_2_O_4_ to multiwalled carbon nanotubes to obtain CuCo_2_O_4_/multiwalled carbon nanotube composite electrode, which obtained a specific capacitance of 1053 F g^−1^ at 2 A g^−1^. The incorporation of carbon nanotubes as conductive additives addressed the problem of the low electronic conductivity of CuCo_2_O_4_. Farid et al. [49] also utilized a hydrothermal strategy to fabricate a CuCo_2_O_4_/RGO composite electrode for constructing solid-state PCs. Their electrochemical characterization further proved that the incorporation of RGO enhanced the electrochemical performance of CuCo_2_O_4_-based devices. Notably, binder-free electrode design is also an efficient strategy for boosting its electronic conductivity [51,52]. For example, Kaverlavani et al. [53] developed a novel strategy combining chemical precipitation and calcination process to grow CuCo_2_O_4_ on 3D nickel foam for fabricating CuCo_2_O_4_-based binder-free electrode. Due to the benefits of a binder-free electrode configuration, the CuCo_2_O_4_-based electrode demonstrated outstanding capacitance, rate performance and cyclic stability. Despite significant efforts to enhance the electronic conductivity of CuCoO_x_-based PCs, their capacitive performance remains below application requirements due to the intrinsically low conductivity of this material.

To overcome this issue, the incorporation of oxygen vacancies has been verified as an efficient strategy for boosting the intrinsic electronic conductivity of metal oxides [1,3,40]. For example, Feng et al. [40] adopted a combined technique of hydrothermal processing and thermal treatment to synthesize CuCo_2_O_4_ enriched by oxygen vacancies for constructing asymmetric supercapacitors with high specific energy density. It was found that the incorporated oxygen vacancies not only enhanced the electronic conductivity of this material but also made it more hydrophilic, which is beneficial for electrolyte access. As a result, the oxygen vacancies in the CuCo_2_O_4_-based electrode demonstrated outstanding capacitive performance. Furtherly, Zhang et al. [42] also combined the hydrothermal method, annealing strategy and chemical vapor deposition to prepare oxygen vacancies incorporated into the Cu_x_Co_3−x_O_4_ electrode material for zinc-ion hybrid supercapacitor application. The obtained Cu_x_Co_3−x_O_4_-based electrode demonstrated a high capacitance value of 1480.7 F g^−1^ (1 A g^−1^), a remarkable rate performance of 88.4% (20 A g^−1^) and a good cyclic stability of 90.1% after 5000 cyclic testing. Density function theory simulation was also carried out to prove that the presence of oxygen vacancies significantly improved the electric conductivity of Cu_x_Co_3−x_O_4_.

However, despite recent progress, the complex preparation procedures and the utilization of many hazardous chemicals for fabricating oxygen-vacancy-incorporated CuCoO_x_ electrodes severely hinder their broad application in MSCs. Therefore, it is extremely important to develop an efficient and versatile manufacturing technique without involving hazardous chemicals for fabricating CuCoO_x_-based MSCs with outstanding electrochemical performance. Recently, nontraditional machining methods like CNCWEDM [2,3,39], micro-ultrasonic machining [54,55,56], ultrasonic micromilling [57] and electrochemical machining [58] have been used widely to fabricate microelectrodes, microchannels and microholes. Among these manufacturing strategies, CNCWEDM is considered a promising process that utilizes the electro-thermal effect to achieve precision machining of various conductive materials and complex structures [59,60]. This machining method has been utilized in a variety of fields, including aerospace components, micro-sensors, mold fabrication, and MSCs [2,61]. Moreover, it is important to note that the CNCWEDM machining parameters such as machining voltage, machining current and machining pulse width could directly tailor the surface characteristics of the metal oxide layer on the workpieces, which is beneficial for creating plentiful active sites for energy storage [1,52,62]. Additionally, the green manufacturing method of CNCWEDM could efficiently prepare various metal oxides and bimetallic oxides in one-step without the utilization of any chemicals, toxic solutions, templates, and additional auxiliary processes. According to our knowledge, there is no research focused on developing oxygen-vacancy-incorporated CuCoO_x_-based binder-free electrodes for MSC applications. Therefore, it is essential to develop the CNCWEDM method and find the relationship between the CNCWEDM machining parameters and the capacitive performance of the machined CuCoO_x_-based MSCs.

Here, oxygen-vacancy-modified CoCuO_x_-based binder-free electrodes were fabricated using a one-step CNCWEDM strategy. The CoCuO_x_ electrodes were machined into different maze-like MSCs with one round (1-CoCuMMSCs-60), two rounds (2-CoCuMMSCs-60) and three rounds (3-CoCuMMSCs-60), respectively, via CNCWEDM with a machining voltage of 60 V. Due to the benefits of narrow electrode fingers, the 3-CoCuMMSCs-60 devices showed the best performance among these three device configurations. Moreover, various CoCuMMSCs with three rounds were machined by different CNCWEDM machining voltages of 60, 80 and 100 V, which are named 3-CoCuMMSCs-60, 3-CoCuMMSCs-80 and 3-CoCuMMSCs-100, respectively. It was found that the machining voltage of CNCWEDM has a strong effect on the morphology and capacitive performance of CoCuMMSCs devices. Impressively, the 3-CoCuMMSCs-100 device achieved the maximum capacitance of 32.8 mF cm^−2^ at 0.15 mA cm^−2^ and worked well at a superhigh scan rate up to 50,000 mV s^−1^. The results prove that the developed CNCWEDM technique facilitated manufacturing of CoCuMMSCs devices with excellent performance by the comprehensive utilization of oxygen-vacancy incorporation, synergistic effect of cobalt and copper oxides, binder-free electrode design, proper device construction and controllable machining voltage. Moreover, it needs to be noted that the developed CNCWEDM technique facilitated manufacturing of CoCuMMSCs devices with excellent performance without the utilization of binders, extra conductive-media, additional current-collectors, hazardous chemicals, inert gas atmosphere, and pre-treatment/post-treatment procedures. The advanced CNCWEDM strategy blazes a new trail for the high-efficiency fabrication of high-performance bimetallic-oxide-based micro-electronics, such as MSCs, intelligent micro-sensors and micro-batteries.

## 2. Experimental Section

### 2.1. Materials

Highly pure Co metal sheets (99.99%) were obtained from Qinghelisheng Metal Materials Company (Xingtai, China). The copper wire for cutting of electrode was supplied by the Suzhou Jinguangxing EDM Machine Tool Parts Operation Department (Suzhou, China). The deionized water (DIW) utilized in this study was supplied by Foshan Putian Yuxin Water Treatment Equipment Company (Foshan, China). A 1.000 mol L^−1^ KOH aqueous solution was obtained from Kell Chemical Technology Company (Wuxi, China).

### 2.2. CoCuMMSCs Preparation

**Figure 1:**
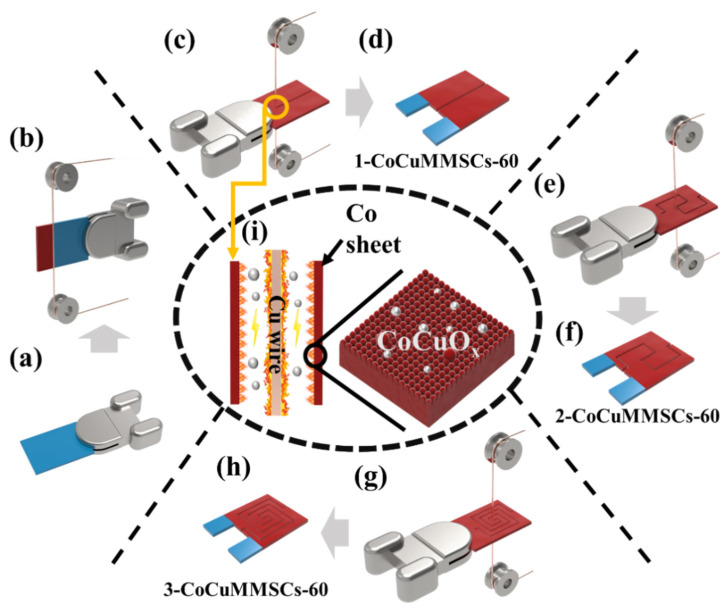
Fabrication procedures of CoCuMMSCs electrodes and devices by CNCWEDM, (**a**) Co sheet before machining, (**b**) Co sheet during CNCWEDM processing, (**c**,**e**,**g**) the patterning process of different structures of CoCuMMSCs, (**d**,**f**,**h**) three structures of CoCuMMSCs and (**i**) the enlarged discharge channel during CNCWEDM.

As demonstrated in Figure 1, the CoCuMMSC electrodes and devices were fabricated by the CNCWEDM method. The experiments were carried out by utilizing a CNCWEDM wire cutting machine called HB400C (Suzhou Sanguang Science & Technology Co., Ltd., Suzhou, China). The CoCuO_x_ binder-free electrodes were prepared by means of CNCWEDM surface treatment of a Co metal sheet using a copper wire as a cutting tool at the 60 V machining voltage. Benefiting from the computer-aided design of the CNCWEDM system, the obtained CoCuO_x_ electrodes were machined into different maze-like MSCs, such as 1-CoCuMMSCs-60 with one round, 2-CoCuMMSCs-60 with two rounds and 3-CoCuMMSCs-60 with three rounds via CNCWEDM with a machining voltage of 60 V (Figure 1c,e,g). Thereafter, various maze-like MSCs of 1-CoCuMMSCs-60, 2-CoCuMMSCs-60 and 3-CoCuMMSCs-60 were obtained. The CNCWEDM technique can be employed by adjusting various process parameters. In order to uncover the effect of CNCWEDM machining voltage on the capacitive behavior of CoCuMMSCs with three rounds, 3-CoCuMMSCs-60, 3-CoCuMMSCs-80 and 3-CoCuMMSCs-100 were manufactured by various CNCWEDM machining voltages of 60, 80, and 100 V. All CNCWEDM processes were performed with a machining current of 2 A, a pulse duration of 12 μs, a duty ratio of 4:1, and the dielectric fluid of deionized water.

### 2.3. Characterization of Electrode Materials

The surface morphologies and microstructure of the CoCuO_x_ electrodes were analyzed by a scanning electron microscope (SEM, TESCAN MIRA LMS, Brno, Czech Republic). The element composition and distribution of the CoCuO_x_ electrode surface were subsequently analyzed using energy-dispersive X-ray spectroscopy (EDS). The crystal structures of CoCuO_x_ binder-free electrodes were studied by X-ray diffraction (XRD, Rigaku Ultima IV, Tokyo, Japan). The element composition, chemical state, and electronic structure of the CoCuO_x_ binder-free electrodes were investigated by X-ray Photoelectron Spectroscopy (XPS, Thermo Scientific K-Alpha, Waltham, MA, USA). The oxygen vacancies presence on the surface of CoCuO_x_ binder-free electrode was verified by Electron Paramagnetic Resonance (EPR) testing, which was carried out by means of a Bruker EMXplus-6/1 instrument obtained from Mannheim, Germany.

### 2.4. Characterization of Electrochemical Properties

The 1-CoCuMMSCs-60, 2-CoCuMMSCs-60, 3-CoCuMMSCs-60, 3-CoCuMMSCs-80 and 3-CoCuMMSCs-100 devices fabricated by the CNCWEDM technique were subjected to a series of electrochemical tests, including a cyclic-voltammetry (CV) test and a galvanostatic charge–discharge (GCD) test, which were performed with a CHI660E electrochemical workstation (CH Instruments, Inc, Shanghai, China). The CV and GCD tests of the CoCuMSCs devices were performed in the voltage window of 0–0.6 V. The electrochemical tests for the CoCuMSCs devices were conducted in 1.000 mol L^−1^ KOH electrolyte.

## 3. Results and Discussion

**Figure 2:**
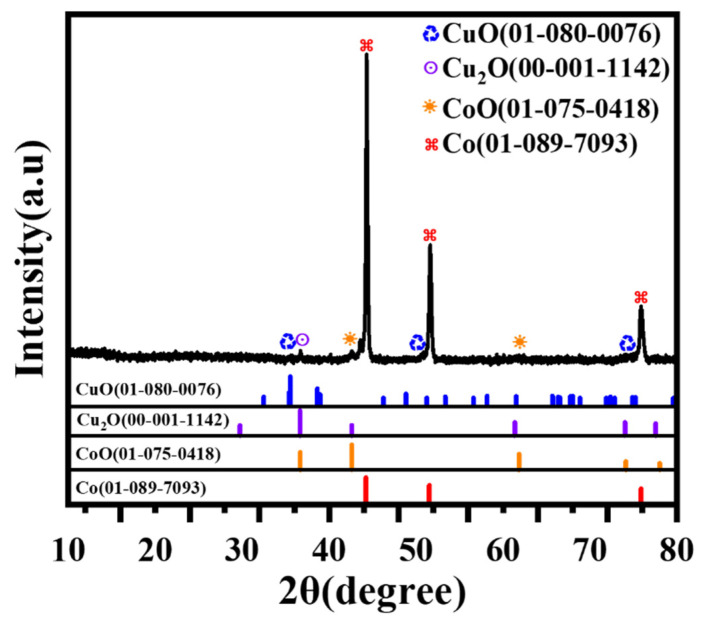
XRD patterns and PDF card numbers for CoCuO_x_ electrodes prepared by CNCWEDM.

Figure 2 illustrates the XRD profile of the CoCuO_x_ electrode. The diffraction pattern exhibits strong peaks at 44.2°, 51.5°, and 75.9°, standing for the (1 1 1), (2 0 0), and (2 2 0) crystal planes of cobalt (JCPDS No. 01-089-7093), respectively [63,64]. Furthermore, diffraction peaks positioned at 42.6°and 61.8°agreed with the (2 0 0) and (2 2 0) crystal planes of CoO. (JCPDS No. 01-075-0418) [64,65]. The results indicated the successful synthesis of CoO particles through the oxidation process under elevated temperatures during the CNCWEDM procedure. Additionally, the diffraction peaks presented at 35.5°, 51.2°, and 74.9° were matched to the (−1 1 1), (1 1 2), and (0 0 4) crystal planes of CuO (JCPDS No. 01-080-0076), respectively [66,67]. The diffraction peak observed at 36.7° could be represented by the (1 1 1) crystal plane of Cu_2_O (JCPDS No. 00-001-1142) [68,69]. Therefore, it was verified that two valence states of copper oxide were incorporated into the workpiece of cobalt metal sheet after CNCWEDM treatment by the machining tool of copper wire. The multiple valence states of copper oxides are beneficial for the improvement in the electronic conductivity of CoCuO_x_ and enhancing its electrochemical behavior. As illustrated in Figure S1, EDS spectra demonstrated evenly distributed cobalt, copper, and oxygen elements on the Co substrate surface after CNCWEDM processing. This outcome further validated that CoCuO_x_ particles were synthesized when machining cobalt metal sheet using copper wire in the CNCWEDM technique.

**Figure 3:**
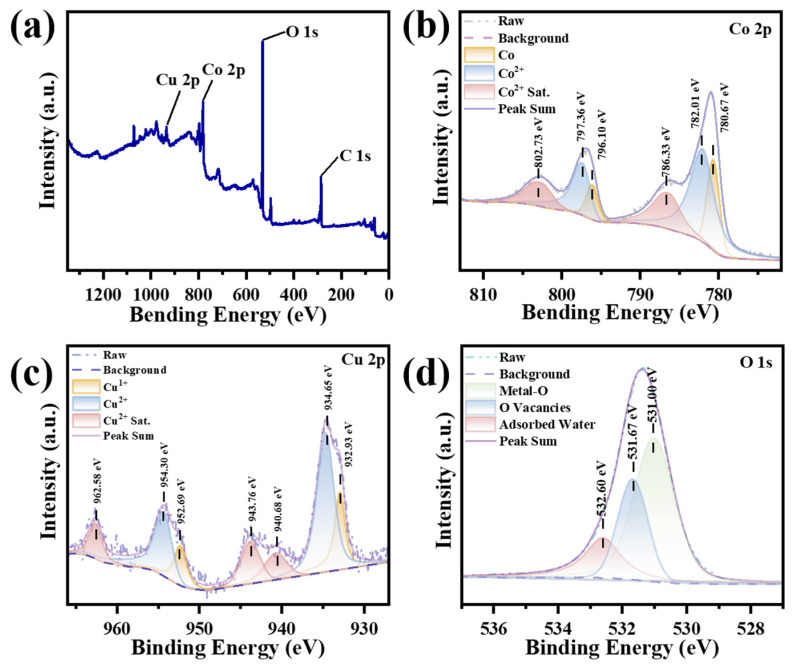
XPS profiles of CoCuO_x_ electrodes: (**a**) survey scan, (**b**) Co 2p, (**c**) Cu 2p, and (**d**) O 1s.

Moreover, XPS was performed to analyze the surface chemical and electronic states of CoCuO_x_ electrode fabricated by the CNCWEDM strategy. Figure 3a–d illustrates the XPS characterization results for the CoCuO_x_ electrode with respect to Co 2p, Cu 2p, and O 1s. All XPS spectra were standardized by C1s at 284.8 eV as a reference standard. Figure 3a presents the full spectrum of the survey of CoCuO_x_ electrode, indicating Co, Cu, and O elements. Figure 3b illustrates two distinct characteristic peaks at 780.7 eV and 796.8 eV, which belonged to Co 2p_3/2_ and Co 2p_1/2_, respectively. Furthermore, the peaks positioned at 780.67 eV and 796.10 eV could belong to Co, while the peaks located at 782.01 eV and 797.36 eV, along with two satellite peaks at 786.33 eV and 802.73 eV, match well with Co^2+^ [70,71,72]. Figure 3c presents the XPS spectrum of Cu 2p. The two strong peaks at 933.8 eV and 953.7 eV correspond to Cu 2p_3_/_2_ and Cu 2p_1_/_2_, respectively. The Cu 2p_3_/_2_ peak could be decomposed into two components at 932.93 eV and 934.65 eV, which stand for Cu^+^ (Cu_2_O) and Cu^2+^ (CuO), respectively. Furthermore, satellite peaks at 943.76 eV, 940.68 eV, and 962.58 eV, which are assigned to Cu^2+^, provide additional confirmation of the Cu^2+^ presence [73,74]. These observations signified the formation of CuO, Cu_2_O and CoO in CoCuO_x_ electrodes, and mixed metal valence of Co^2+^, Cu^+^ and Cu^2+^ is beneficial for the efficient energy storage. Figure 3d shows the O 1s profile, which can be decomposed into three standard peaks located at 531, 531.67, and 532.6 eV. These peaks stand for metal–oxygen bonding, oxygen vacancies, and surface-adsorbed water, respectively [75,76,77,78]. The presence of oxygen vacancies was subsequently confirmed through an EPR test (Figure S2). It was found that the CoCuO_x_ electrode obtained g = 2.0055, which is located near the standard value of free electrons (g = 2.0023) [79,80]. Therefore, the EPR characterized result indicated that the oxygen vacancies could be effectively generated through a one-step CNCWEDM treatment. The existence of oxygen vacancies is beneficial for providing sufficient electrochemical active sites of CoCuO_x_ binder-free electrode for charge storage and promote its electrochemical reaction [81,82].

**Figure 4:**
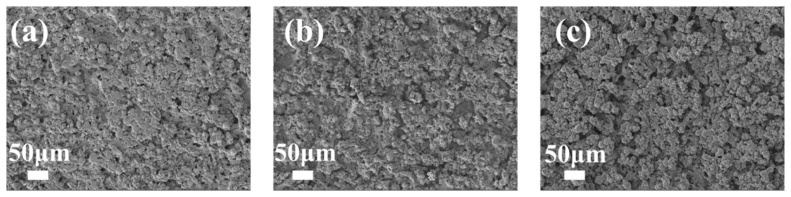
SEM of CNCWEDM after different machining voltages: (**a**) 60 V, (**b**) 80 V, and (**c**) 100 V.

Subsequently, SEM observations were utilized to examine alterations in the surface morphology of CoCuO_x_ binder-free electrode under varying machining voltages adjusted by CNCWEDM. Figure 4 presents a visual representation of the surface characteristics of CoCuO_x_ binder-free electrodes created by various CNCWEDM machining voltages of 60 V, 80 V, and 100 V. In addition, low-magnification SEM images with scale bars of 10 μm are provided in the Supplementary Materials (Figure S3) to further illustrate the overall surface morphology. It is seen that the polished cobalt metal sheets with smooth surface have been transferred into very rough surface covered by CoCuO_x_ particles after treatment by CNCWEDM. The CoCuO_x_ binder-free electrodes with enhanced surface roughness were able to provide increasing specific surface area and plentiful electrochemical active sites for speeding up their Faradaic reaction and boosting their capacitive performance. Another important finding is that the CoCuO_x_ binder-free electrodes prepared by machining voltages of 60 V and 80 V showed relatively denser and smoother surface characteristic compared to that achieved by a machining voltage of 100 V. The rough and porous structure of CoCuO_x_ binder-free electrodes prepared by a machining voltage of 100 V is beneficial for speeding up the ionic and electronic transferred efficiency and thus improving their electrochemical performance.

**Figure 5:**
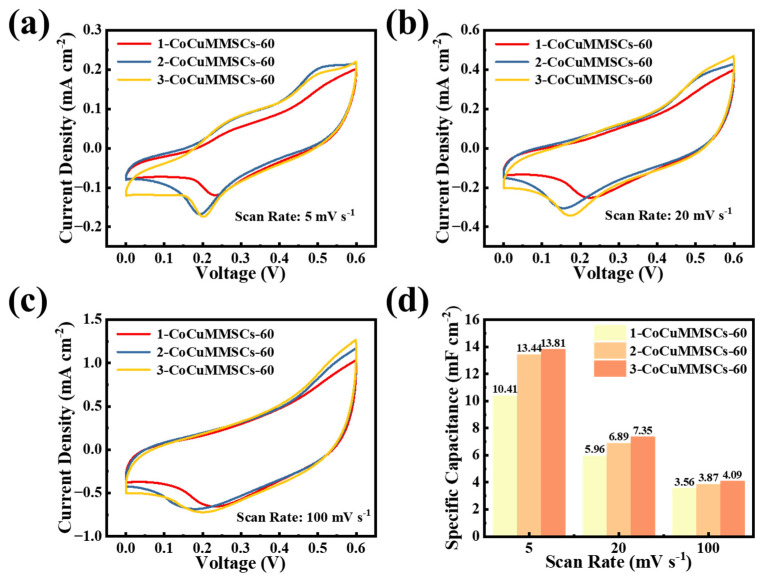
(**a**–**c**) The CV curves of 1-CoCuMMSCs-60, 2-CoCuMMSCs-60, and 3-CoCuMMSCs-60 recorded, (**d**) the specific capacitance values derived from the CV curves at scan rates of 5, 20, and 100 mV s^−1^.

**Figure 6:**
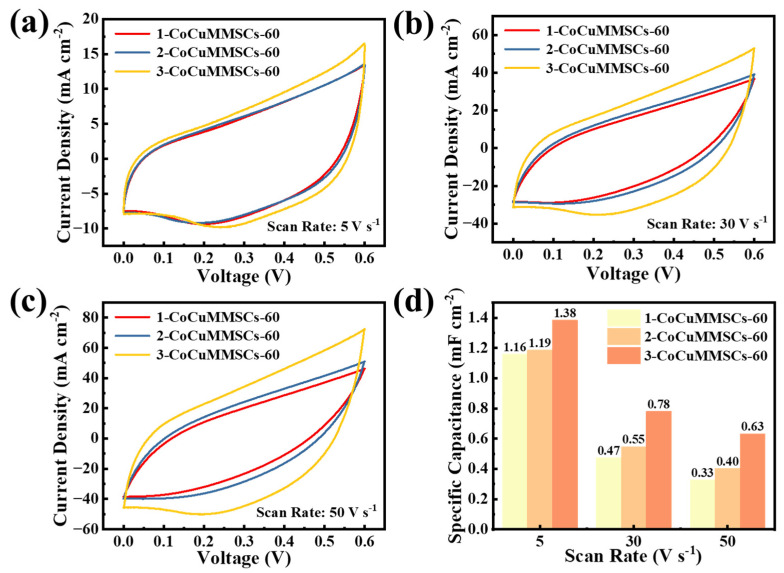
The CV curves of 1-CoCuMMSCs-60, 2-CoCuMMSCs-60, and 3-CoCuMMSCs-60 recorded at (**a**) 5 V s^−1^, (**b**) 30 V s^−1^, (**c**) 50 V s^−1^, (**d**) the specific capacitance values derived from the CV curves at scan rates of 5, 30, and 50 V s^−1^.

**Figure 7:**
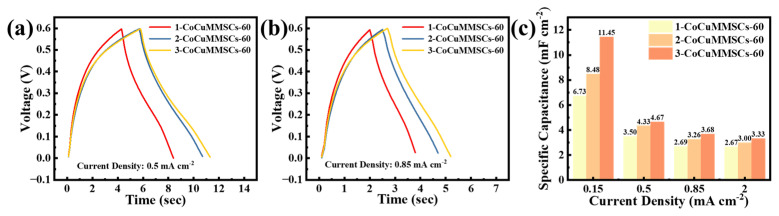
GCD curves for 1-CoCuMMSCs-60, 2-CoCuMMSCs-60, and 3-CoCuMMSCs-60 at (**a**) 0.5 mA cm^−2^ and (**b**) 0.85 mA cm^−2^, (**c**) the capacitance value derived from GCD curves.

It is important to note that the geometric shape of planar MSCs plays an important role in their electrochemical performance. Benefiting from the computer-aided design system of CNCWEDM, CoCuMMSCs with different maze-like configurations, including 1-CoCuMMSCs-60, 2-CoCuMMSCs-60, and 3-CoCuMMSCs-60, were designed and manufactured via this efficient fabrication strategy of CNCWEDM with the same machining voltage of 60 V. Subsequently, 1-CoCuMMSCs-60, 2-CoCuMMSCs-60, and 3-CoCuMMSCs-60 devices were characterized by CV and GCD examinations. Figure 5 shows the CV-examined results of 1-CoCuMMSCs-60, 2-CoCuMMSCs-60, and 3-CoCuMMSCs-60 at 5 mV s^−1^ (Figure 5a), 20 mV s^−1^ (Figure 5b) and 100 mV s^−1^ (Figure 5c). It was found that 3-CoCuMMSCs-60 with the smallest finger width obtained the largest CV area, whereas the 1-CoCuMMSCs-60 with the largest finger width obtained the smallest CV area. As a result, Figure 5d demonstrates that 3-CoCuMMSCs-60 obtained the largest area-normalized capacitance of 13.81 mF cm^−2^ (at 5 mV s^−1^), whereas 1-CoCuMMSCs-60 obtained the smallest area-normalized capacitance of 10.41 mF cm^−2^ (at 5 mV s^−1^). The 3-CoCuMMSCs-60 device obtained the best capacitive performance because this maze-like-designed 3-CoCuMMSCs-60 with the smallest finger width facilitates shortening the ion movement distance and thus enhancing the whole device charge storing efficiency [83,84]. Moreover, the electrostatic field and potential distributions of 1-CoCuMMSCs-60, 2-CoCuMMSCs-60, and 3-CoCuMMSCs-60 were simulated (Figure S4). The simulation results demonstrated that the 3-CoCuMMSCs-60 device obtained the highest electric field density, which is beneficial for accelerating the ions’ transport rate and boosting the capacitive performance. Figure 6 shows the CV-examined results of 1-CoCuMMSCs-60, 2-CoCuMMSCs-60, and 3-CoCuMMSCs-60 at extremely high-testing-rate conditions of 5 V s^−1^ (Figure 6a), 30 V s^−1^ (Figure 6b) and 50 V s^−1^ (Figure 6c). It is clearly observed that all the maze-like CoCuO_x_-based MSCs manufactured by this new strategy of CNCWEDM show remarkable capacitive characteristics, which opens a new avenue for fabricating high power-density MSCs. This excellent capacitive behavior achieved by CoCuMMSCs fabricated by CNCWEDM can be attributed to the binder-free CoCuO_x_ electrode configuration, mixed metal valence of Co^2+^, Cu^+^ and Cu^2+^, and the incorporation of oxygen vacancies in CoCuO_x_, which are conducive to accelerating the electrons’/ions’ transporting efficiency [85,86]. Moreover, it could be concluded that the CoCuMMSCs devices showed increasing current response with the decrease in finger width. This is because the smaller finger width allows for better ion transporting efficiency. As a result, 3-CoCuMMSCs-60 obtained largest area-normalized capacitance, whereas 1-CoCuMMSCs-60 obtained the smallest one (Figure 6d). This phenomenon observed from the extremely high-testing-rate conditions is consistent with that obtained at small scan rates (Figure 5). Furthermore, Figure 7 provides a comparison of the GCD results for 1- CoCuMMSCs-60, 2- CoCuMMSCs-60, and 3-CoCuMMSCs-60 at 0.5 mA cm^−2^ (Figure 7a) and 0.85 mA cm^−2^ (Figure 7b). It could be observed that 3-CoCuMMSCs-60 with the smallest finger width exhibited the longest charging and discharging times, whereas the 1-CoCuMMSCs-60 with largest finger width showed the shortest charging and discharging times at both examined conditions of 0.5 mA cm^−2^ and 0.85 mA cm^−2^. Therefore, the 3-CoCuMMSCs-60 device obtained the largest area-normalized capacitance of 11.45 mF cm^−2^ (at 0.15 mA cm^−2^) among these three devices with various configurations. This phenomenon illustrated in Figure 7 showed good agreement with that observed from CV examination (Figure 5 and Figure 6).

**Figure 8:**
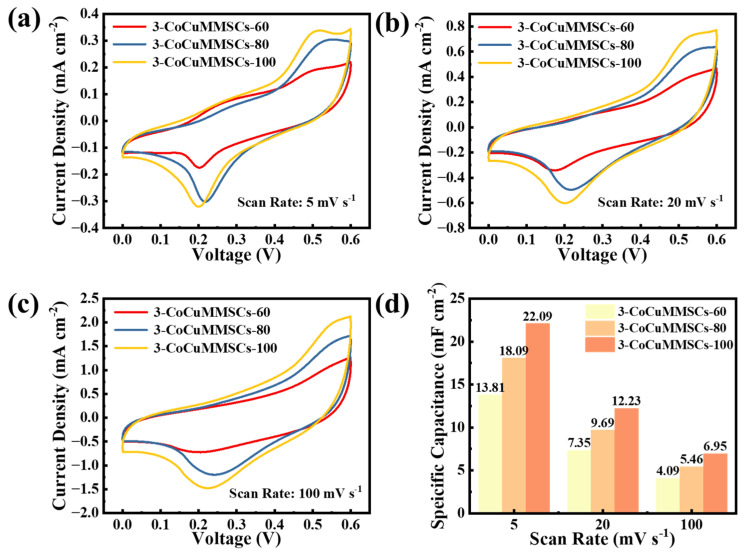
The CV curves of 3-CoCuMMSCs-60, 3-CoCuMMSCs-80, and 3-CoCuMMSCs-100 at scan rates: (**a**) 5 mV s^−1^, (**b**) 20 mV s^−1^, and (**c**) 100 mV s^−1^, (**d**) the specific capacitance values derived from the CV curves at scan rates of 5, 20, and 100 mV s^−1^.

Apart from customizing the geometric shape of CoCuMMSCs, this novel CNCWEDM technique can customize the surface morphologies of CoCuO_x_ electrodes by simply adjusting the machining voltage. Therefore, the 3-CoCuMMSCs-60, 3-CoCuMMSCs-80, and 3-CoCuMMSCs-100 devices were obtained by different CNCWEDM machining voltages of 60, 80 and 100 V, respectively. Figure 8a–c depicts the CV characterization results for 3-CoCuMMSCs-60, 3-CoCuMMSCs-80, and 3-CoCuMMSCs-100. Based on the CV analysis, it can be observed that as the preparation voltage increases, the integral area of the CV curve becomes larger, which indicates enhancing capacitive performance. The area-normalized capacitances of 3-CoCuMMSCs-60, 3-CoCuMMSCs-80, and 3-CoCuMMSCs-100 devices were obtained from the CV data (Figure 8d). From the comparison of the data for 3-CoCuMMSCs-60 and 3-CoCuMMSCs-80, it is noteworthy that 3-CoCuMMSCs-100 demonstrated the maximum area-normalized capacitances at all examined conditions. The 3-CoCuMMSCs-100 device showed an area-normalized capacitance of 22.09 mF cm^−2^ (5 mV s^−1^), which is around 1.2 times that of 3-CoCuMMSCs-80 and 1.6 times that of 3-CoCuMMSCs-60.

**Figure 9:**
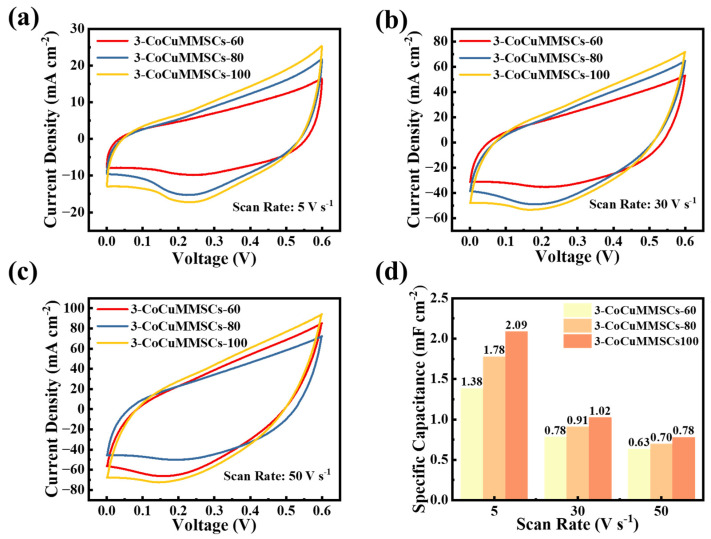
The CV curves of 3-CoCuMMSCs-60, 3-CoCuMMSCs-80, and 3-CoCuMMSCs-100 at (**a**) 5 V s^−1^, (**b**) 30 V s^−1^, and (**c**) 50 V s^−1^, (**d**) the specific capacitance values derived from the CV curves at scan rates of 5, 30, and 50 V s^−1^.

**Figure 10:**
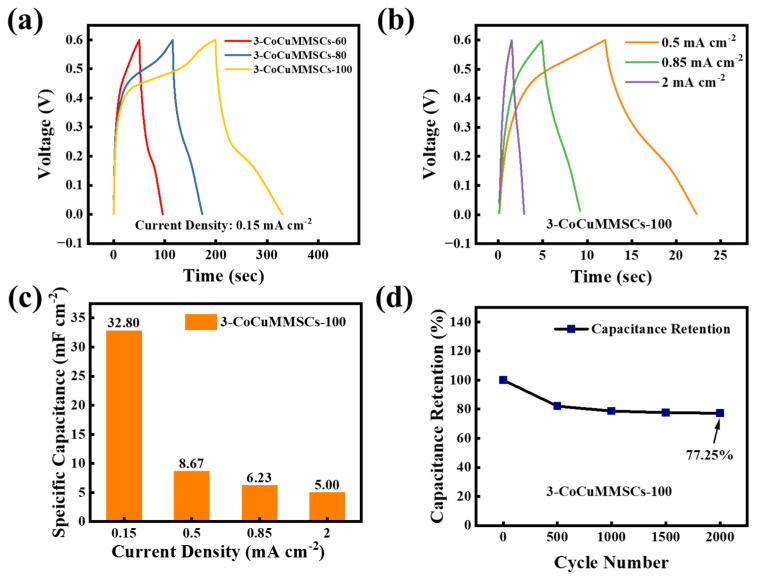
(**a**) GCD for CoCuMMSCs at 0.15 mA cm^−2^, (**b**) GCD of 3-CoCuMMSCs-100 from 0.5 to 2 mA cm^−2^. (**c**) The specific capacitance at different current densities for 3-CoCuMMSCs-100. (**d**) Cycling stability of 3-CoCuMMSCs-100 at 500 mV s^−1^.

Furthermore, the fast charging/discharging characteristic is one of the most important parameters for MSCs. Therefore, the 3-CoCuMMSCs-60, 3-CoCuMMSCs-80, and 3-CoCuMMSCs-100 devices were characterized at extremely high testing conditions of 5 V s^−1^ (Figure 9a), 30 V s^−1^ (Figure 9b) and 50 V s^−1^ (Figure 9c). It is clearly observed that all the maze-like CoCuO_x_-based MSCs manufactured by this novel CNCWEDM method with various machining voltages depict good capacitive characteristics, which opens a new avenue for fabricating fast charging/discharging MSCs. This excellent capacitive behavior achieved by 3-CoCuMMSCs-60, 3-CoCuMMSCs-80, and 3-CoCuMMSCs-100 devices fabricated by CNCWEDM can be attributed to the following advantages, including the binder-free CoCuO_x_ electrode configuration, and CoCuO_x_ bimetallic oxide with mixed-metal ions of Co^2+^, Cu^+^ and Cu^2+^, as well as CoCuO_x_ particles with the incorporation of oxygen vacancies, which greatly accelerate their energy storage efficiency [85,86]. Moreover, it could be concluded that the CoCuMMSCs showed increasing current response with the increase in machining voltage. This is because the higher machining voltage facilitates the synthesis of CoCuO_x_ with porous structures, providing sufficient electrochemical active sites for ion access. As a result, 3-CoCuMMSCs-100 obtained the largest area-normalized capacitance, whereas 3-CoCuMMSCs-60 gained the smallest one (Figure 9d). This phenomenon observed from the extremely high-testing-rate conditions is consistent with that obtained at small scan rates (Figure 8). Furthermore, Figure 10 and Figure S5 provide the GCD results of 3-CoCuMMSCs-60, 3-CoCuMMSCs-80 and 3-CoCuMMSCs-100 at 0.15 mA cm^−2^ (Figure 10a), 0.5 mA cm^−2^ (Figure S5a) and 0.85 mA cm^−2^ (Figure S5b). It can be observed that 3-CoCuMMSCs-100 machined by the highest machining voltage of 100 V exhibited the longest charging and discharging times, whereas 3-CoCuMMSCs-60 cut by the lowest machining voltage of 60 V showed the shortest charging and discharging times at both examined conditions of 0.5 mA cm^−2^ and 0.85 mA cm^−2^. It was found that the 3-CoCuMMSCs-100 device fabricated by CNCWEDM obtained the largest area-normalized capacitance of 32.8 mF cm^−2^ (at 0.15 mA cm^−2^), which is superior to previous reported values, including Ni/MnO_2_ MSCs (4.15 mF cm^−2^) prepared via the stamp-assisted printing technique [87], graphene/V_2_O_5_ composite-based MSCs (3.29 mF cm^−2^) fabricated via oxygen plasma etching, masking and depositing strategies [88], graphene/CuO composite-based MSCs (6.45 mF cm^−2^) manufactured by a laser writing technique [89], and NiCo_2_O_4_ bimetallic-oxide-based MSCs (18.8 mF cm^−2^) obtained by atomic layer deposition, hydrothermal, and electron-beam evaporation methods [90]. In addition, it is noted that these reported MSCs manufacturing techniques (like stamp-assisted printing, hydrothermal method, etc.) in the literature not only require the use of toxic and harmful solvents/chemicals, but also suffer from the problem of complex device preparation procedures. Meanwhile, to further evaluate the electrochemical durability under high-rate conditions, the cycling performance of the 3-CoCuMMSCs-100 device was also investigated. As shown in Figure 10d, the device retains 77.25% of its initial capacitance after 2000 cycles at a scan rate of 500 mV s^−1^, indicating good cycling stability. Therefore, this developed one-step CNCWEDM technique for fabricating CoCuMMSCs greatly simplifies the MSCs processing procedures and makes the manufacturing processing more environmentally friendly.

## 4. Conclusions

In this work, oxygen-vacancy-modified CoCuO_x_-based binder-free electrodes were fabricated using a one-step CNCWEDM strategy. Moreover, this approach enabled the fabrication of CoCuO_x_-based maze-like MSCs with designable electrochemical performance, which could be simply controlled by their geometric shape and machining voltage. Impressively, theoretical simulation demonstrated that the 3-CoCuMMSCs-60 device with the smallest finger width obtained the highest electric field density, which is beneficial for accelerating the ions’ transport rate and boosting the capacitive performance. Moreover, it has been verified that the capacitive performance of CoCuMMSCs was successfully customized by the machining voltage of CNCWEDM. Moreover, the 3-CoCuMMSCs-100 fabricated by a machining voltage of 100 V achieved the maximum capacitance of 32.8 mF cm^−2^ at 0.15 mA cm^−2^ and worked well at a superhigh scan rate up to 50,000 mV s^−1^, which is more than two orders of magnitude larger than previously reported testing scan rates. These results proved that the developed CNCWEDM technique facilitates manufacturing CoCuMMSCs devices with excellent performance by the comprehensive utilization of oxygen-vacancy incorporation, cooperative effect of cobalt oxides and copper oxides, binder-free electrode design, proper device construction and controllable machining voltage. This advanced CNCWEDM strategy blazes a new trail for the high-efficiency fabrication of high-performance bimetallic-oxide-based micro-electronics, such as MSCs, intelligent micro-sensors and micro-batteries.

## Data Availability

The original contributions presented in the work are included in the article, further inquiries can be directed to the corresponding author.

## Supplementary Materials

The following supporting information can be downloaded at: https://www.mdpi.com/article/10.3390/mi17050516/s1, Figure S1: EDS mapping of individual elements for the CoCuO_x_ electrode: (a) Co, (b) Cu, and (c) O; Figure S2: EPR spectrum of the CoCuO_x_ electrode; Figure S3: SEM (scale bar: 10 μm) of CNCWEDM processed at different machining voltages: (a) 60 V, (b) 80 V, and (c) 100 V;Figure S4: Electrostatic field and potential distributions at 0.6 V for different electrode structures. (a–c) Electric field intensity distributions for electrode structures 1-CoCuMMSCs60, 2-CoCuMMSCs60, and 3-CoCuMMSCs60, respectively. (d–f) Potential distribution maps for the corresponding structures; Figure S5: GCD for CoCuMMSCs at (a) 0.5 mA cm^−2^ and (b) 0.85 mA cm^−2^.

## References

[1] Chen R., Lv S., Xu Z., Qin J., Xu Y., Shu Z., Wang B., Qu M., Wang W., Zhitomirsky I. (2025). Unraveling the Effect of Electric Discharge Machining Current on the Fabrication of 3D Mo-Doped VO_0.2_ Integrated Microsupercapacitors. Adv. Eng. Mater..

[2] Xu Y., Deng P., Chen R., Xie W., Xu Z., Yang Y., Liu D., Huang F., Zhuang Z., Zhitomirsky I. (2023). Electric discharge direct writing of 3D Mo-MoOx pseudocapacitive micro-supercapacitors with designable patterns. Ceram. Int..

[3] Chen R., Xu Z., Xu Y., Tao Z., Jiang M., Zhitomirsky I., Liu D., Wang J., He J., Yang Y. (2025). 3D binderless CuMoO_x_ ceramic bimetallic oxides based microsupercapacitors with tailorable performance manufactured by one-step direct electric discharge writing. J. Energy Storage.

[4] Liu D., Xie W., Xu Z., Deng P., Wu Z., Zhitomirsky I., Wang W., Chen R., Zhou L., Xu Y. (2023). Fabrication of Ni-Cr-FeO_x_ ceramic supercapacitor electrodes and devices by one-step electric discharge ablation. J. Energy Storage.

[5] Chen R., Xu Z., Xie W., Deng P., Xu Y., Xu L., Zhang G., Yang Y., Xie G., Zhitomirsky I. (2023). Fabrication of Fe–Fe_1−*x*_ O based 3D coplanar microsupercapacitors by electric discharge rusting of pure iron substrates. RSC Adv..

[6] El-Kady M.F., Kaner R.B. (2013). Scalable fabrication of high-power graphene micro-supercapacitors for flexible and on-chip energy storage. Nat. Commun..

[7] Beidaghi M., Gogotsi Y. (2014). Capacitive energy storage in micro-scale devices: Recent advances in design and fabrication of micro-supercapacitors. Energy Environ. Sci..

[8] Hu H., Pei Z., Ye C. (2015). Recent advances in designing and fabrication of planar micro-supercapacitors for on-chip energy storage. Energy Storage Mater..

[9] Qi D., Liu Y., Liu Z., Zhang L., Chen X. (2017). Design of Architectures and Materials in In-Plane Micro-supercapacitors: Current Status and Future Challenges. Adv. Mater..

[10] Wang J., Li F., Zhu F., Schmidt O.G. (2019). Recent Progress in Micro-Supercapacitor Design, Integration, and Functionalization. Small Methods.

[11] Li L., Fu C., Lou Z., Chen S., Han W., Jiang K., Chen D., Shen G. (2017). Flexible planar concentric circular micro-supercapacitor arrays for wearable gas sensing application. Nano Energy.

[12] Shen D., Zou G., Liu L., Zhao W., Wu A., Duley W.W., Zhou Y.N. (2018). Scalable High-Performance Ultraminiature Graphene Micro-Supercapacitors by a Hybrid Technique Combining Direct Writing and Controllable Microdroplet Transfer. ACS Appl. Mater. Interfaces.

[13] Zheng S., Li Z., Wu Z.-S., Dong Y., Zhou F., Wang S., Fu Q., Sun C., Guo L., Bao X. (2017). High Packing Density Unidirectional Arrays of Vertically Aligned Graphene with Enhanced Areal Capacitance for High-Power MicroSupercapacitors. ACS Nano.

[14] Yang Z., Ren J., Zhang Z., Chen X., Guan G., Qiu L., Zhang Y., Peng H. (2014). Recent Advancement of Nanostructured Carbon for Energy Applications. Chem. Rev..

[15] Davies A., Yu A. (2011). Material advancements in supercapacitors: From activated carbon to carbon nanotube and graphene. Can. J. Chem. Eng..

[16] Yang Z. (2019). Carbon nanotube- and graphene-based nanomaterials and applications in high-voltage supercapacitor: A review. Carbon.

[17] Gu W., Yushin G. (2013). Review of nanostructured carbon materials for electrochemical capacitor applications: Advantages and limitations of activated carbon, carbidederived carbon, zeolitetemplated carbon, carbon aerogels, carbon nanotubes, onionlike carbon, and graphene. Wiley Interdiscip. Rev. Energy Environ..

[18] Siwal S.S., Zhang Q., Devi N., Thakur V.K. (2020). Carbon-Based Polymer Nanocomposite for High-Performance Energy Storage Applications. Polymers.

[19] Ran F. (2018). Recent progress in carbon-based nanoarchitectures for advanced supercapacitors. Adv. Compos. Hybrid Mater..

[20] Lyu L., Hooch Antink W., Kim Y.S., Kim C.W., Hyeon T., Piao Y. (2021). Recent Development of Flexible and Stretchable Supercapacitors Using Transition Metal Compounds as Electrode Materials. Small.

[21] Naskar P., Maiti A., Chakraborty P., Kundu D., Biswas B., Banerjee A. (2021). Chemical supercapacitors: A review focusing on metallic compounds and conducting polymers. J. Mater. Chem. A.

[22] Sahoo S., Kumar R., Joanni E., Singh R.K., Shim J.-J. (2022). Advances in pseudocapacitive and battery-like electrode materials for high performance supercapacitors. J. Mater. Chem. A.

[23] Sumdani M.G., Islam M.R., Yahaya A.N.A., Safie S.I. (2022). Recent advancements in synthesis, properties, and applications of conductive polymers for electrochemical energy storage devices: A review. Polym. Eng. Sci..

[24] Hong X., Liu Y., Li Y., Wang X., Fu J., Wang X. (2020). Application Progress of Polyaniline, Polypyrrole and Polythiophene in Lithium-Sulfur Batteries. Polymers.

[25] Snook G.A., Kao P., Best A.S. (2011). Conducting-polymer-based supercapacitor devices and electrodes. J. Power Sources.

[26] Rui X., Tan H., Yan Q. (2014). Nanostructured metal sulfides for energy storage. Nanoscale.

[27] Chandrasekaran S., Yao L., Deng L., Bowen C., Zhang Y., Chen S., Lin Z., Peng F., Zhang P. (2019). Recent advances in metal sulfides: From controlled fabrication to electrocatalytic, photocatalytic and photoelectrochemical water splitting and beyond. Chem. Soc. Rev..

[28] Xu X., Liu W., Kim Y., Cho J. (2014). Nanostructured transition metal sulfides for lithium ion batteries: Progress and challenges. Nano Today.

[29] Barik R., Ingole P.P. (2020). Challenges and prospects of metal sulfide materials for supercapacitors. Curr. Opin. Electrochem..

[30] Duan H., Lu J., Li S., Zhang Y., Hu W., Zhu R., Pang H. (2022). Formation of conductive MOF@Metal oxide micro-nano composites via facile self-assembly for high-performance supercapacitors. Mater. Today Chem..

[31] Shaheen I., Hussain I., Zahra T., Javed M.S., Shah S.S.A., Khan K., Hanif M.B., Assiri M.A., Said Z., Arifeen W.U. (2023). Recent advancements in metal oxides for energy storage materials: Design, classification, and electrodes configuration of supercapacitor. J. Energy Storage.

[32] Jayakumar S., Santhosh P.C., Mohideen M.M., Radhamani A.V. (2024). A comprehensive review of metal oxides (RuO_2_, Co_3_O_4_, MnO_2_ and NiO) for supercapacitor applications and global market trends. J. Alloys Compd..

[33] Shaheen N., Aadil M., Zulfiqar S., Sabeeh H., Agboola P.O., Warsi M.F., Aboud M.F.A., Shakir I. (2021). Fabrication of different conductive matrix supported binary metal oxides for supercapacitors applications. Ceram. Int..

[34] Surya K., Michael M. (2022). Pseudocapacitive binary metal oxide NiMn_2_O_4_ nanoparticles as an electrode for high-powered hybrid supercapacitors. J. Mater. Sci. Mater. Electron..

[35] Samage A., Kuppe P., Halakarni M., Ganesan B.K., Kamath S.V., Yoon H., Kotrappanavar N.S. (2024). Room temperature and rapid synthesis of ZnMn_2_O_4_ nanostructured spinel using deep eutectic solvent for high energy asymmetric supercapacitors. J. Energy Storage.

[36] Ahmad R., Sohail A., Altaf U., Farooq J., Mir A., Aalim M., Majeed A., Shah M. (2023). Binary nickel cobalt oxide (NixCo_3−x_O_4_) nanostructures as stable and high-energy density asymmetric supercapacitor electrode material. Mater. Chem. Phys..

[37] Murugesan M., Nallamuthu N., Ranjithkumar R., Krishnakumar M., Devendran P., Ramesh K. (2023). Synthesis and electrochemical investigation of hetero bimetallic complexes CoMn2O4 micro rods for novel supercapacitor electrode. Electron. Mater. Lett..

[38] Jadhavar A., Shelke N.T., Yewale M., Kadam R., Kadam S., Shin D. (2023). Hydrothermal synthesis of cobalt vanadium oxide (Co_3_V_2_O_8_) hexagonal disc for high-performance supercapacitors. Surf. Interfaces.

[39] Yang S., Chen R., Huang F., Wang W., Zhitomirsky I. (2024). One-Step Fabrication of 2.5 D CuMoO_x_ Interdigital Microelectrodes Using Numerically Controlled Electric Discharge Machining for Coplanar Micro-Supercapacitors. Micromachines.

[40] Feng Y., Liu W., Wang Y., Gao W., Li J., Liu K., Wang X., Jiang J. (2020). Oxygen vacancies enhance supercapacitive performance of CuCo_2_O_4_ in high-energy-density asymmetric supercapacitors. J. Power Sources.

[41] Azzou K.A.K., Terbouche A., Ait Ramdane-Terbouche C., Bataille T., Hauchard D., Mezaoui D. (2024). Supercapacitor electrode based on ternary activated carbon/CuCoO_2_ hybrid material. Mater. Chem. Phys..

[42] Zhang X., Akkinepally B., Han K., Jelani M., Javed M.S., Khan S., Hussain I., Hassan A.M., Alshgari R.A., Mushab M. (2023). Vacancy and surface modulation engineering of CuxCo_3_-xO_4_ nanowires as an advanced cathode for zinc-ion hybrid supercapacitors. J. Energy Storage.

[43] Feng Y., Sun L., Qi Z., Zhang Y., Wang G., Gao W., Liu W. (2023). Cationic and anionic defect decoration of CoO through Cu dopants and oxygen vacancy for a High-Performance supercapacitor. J. Colloid Interface Sci..

[44] Fan L., Zhang X., Chen X., Tang S., Xu X., Zhu L., Zhang Q. (2025). Study of phosphorization effect on urchin-like CuCo_2_O_4_ cathodes for high-performance supercapacitors. J. Alloys Compd..

[45] Kadam V., Patil V., Bhosale R., Mujawar S., Torane A., Kadam L. (2025). Development of binary rose-like CuCo_2_O_4_ nanoarchitecture via novel chemical route for electrochemical supercapacitor application. Mater. Sci. Eng. B.

[46] Ensafi A.A., Moosavifard S.E., Rezaei B., Kaverlavani S.K. (2018). Engineering onion-like nanoporous CuCo_2_O_4_ hollow spheres derived from bimetal–organic frameworks for high-performance asymmetric supercapacitors. J. Mater. Chem. A.

[47] Verma S., Pandey V.K., Verma B. (2022). Facile synthesis of graphene oxide-polyaniline-copper cobaltite (GO/PANI/CuCo_2_O_4_) hybrid nanocomposite for supercapacitor applications. Synth. Met..

[48] Qi C., Liu Y., Wang S., Du S., Li S., Yang W., Yang H., Wang L., Li Y. (2024). Facile synthesis of CuCo_2_O_4_/N-deficient-carbon nitride heterostructure material for high-performance asymmetric supercapacitors. J. Energy Storage.

[49] Farid H.M.T., Gouadria S., Al-Moayid S., Algarni H., Ansari M.Z., Ali H.E. (2023). Facile synthesis of CuCo_2_O_4_ spinel with rGO nanocomposite via hydrothermal approach for solid state supercapacitor application. J. Energy Storage.

[50] Bhagwan J., Han J.I. (2023). CuCo_2_O_4_ nanoplates anchored to multiwall carbon nanotubes as an enhanced supercapacitive performance. J. Energy Storage.

[51] Yang S., Chen R., Huang F., Wang W., Zhitomirsky I. (2024). 3D Binder-Free Mo@ CoO Electrodes Directly Manufactured in One Step via Electric Discharge Machining for In-Plane Microsupercapacitor Application. Micromachines.

[52] Chen R., Qin J., Xu Z., Lv S., Tao Z., He J., Zhou P., Shu Z., Zhuang Z., Wang W. (2024). The impact of processing voltage of wire electric discharge machining on the performance of Mo doped V–VO 0.2 based Archimedean micro-supercapacitors. RSC Adv..

[53] Kamari Kaverlavani S., Abbasi L., Mishra Y.K., Hosseini S.Y., Liavali M.N., Moosavifard S.E. (2022). Tunable fabrication of hollow nano sword-like CuCo_2_O_4_ derived from bimetal–organic frameworks as binder-free electrodes. ACS Sustain. Chem. Eng..

[54] He J., Guo Z., Lian H., Wang J., Liu J., Chen X. (2019). Study on manufacturing quality of micro-ultrasonic machining with force control. Int. J. Adv. Manuf. Technol..

[55] Lian H., Deng C., Zhang L., Mo Y., He J., Guo Z. (2023). Fabrication of microchannels through template-based electrophoretically assisted micro-ultrasonic machining. Int. J. Adv. Manuf. Technol..

[56] Lian H., Zhang L., Chen X., Deng C., Mo Y. (2023). Design of a Template-Based Electrophoretically Assisted Micro-Ultrasonic Machining Micro-Channel Machine Tool and Its Machining Experiment. Micromachines.

[57] He J., Guo Z., Lian H., Wang J., Chen X., Liu J. (2020). Improving the machining quality of micro structures by using electrophoresis-assisted ultrasonic micromilling machining. Int. J. Precis. Eng. Manuf.-Green Technol..

[58] He J., Wang Z., Wang J., Liang H., Lian H. (2023). Investigation of the microhole arrays generated by masked jet electrochemical machining with polyaluminum chloride electrolyte. Precis. Eng..

[59] Sivapirakasam S.P., Mathew J., Surianarayanan M. (2011). Multi-attribute decision making for green electrical discharge machining. Expert Syst. Appl..

[60] Sai Ram J., Jeavudeen S., Mouda P.A., Ahamed N. (2026). The role of various dielectrics used in EDM process and their environmental, health, and safety issues. Mater. Today Proc..

[61] Ulhakim M.T., Sukarman, Khoirudin, Mulyadi D., Susilo H., Rohman, Setyo M. (2025). Electrical Discharge Machining: Recent Advances and Future Trends in Modeling, Optimization, and Sustainability. Int. J. Lightweight Mater. Manuf..

[62] Chen R., Qin J., Chen Z., Yang Z., Liu J., Zheng B., Wang W., Zhitomirsky I., Zhan Z., He J. (2026). A versatile strategy of wire electric discharge machining for one-step fabrication of molybdenum-based micro-supercapacitors: From MoO_x_ to CuMoO_x_. J. Mater. Sci..

[63] Li Z., Shao M., Zhou L., Zhang R., Zhang C., Han J., Wei M., Evans D.G., Duan X. (2016). A flexible all-solid-state micro-supercapacitor based on hierarchical CuO@ layered double hydroxide core–shell nanoarrays. Nano Energy.

[64] Cai J., Chen Y., Song H., Hou L., Li Z. (2020). MOF derived C/Co@C with a “one-way-valve”-like graphitic carbon layer for selective semi-hydrogenation of aromatic alkynes. Carbon.

[65] Logacheva V.A., Lukin A.N., Afonin N.N., Serbin O.V. (2019). Synthesis and Optical Properties of Cobalt-Modified Titanium Oxide Films. Opt. Spectrosc..

[66] John S., Vadla S.S., Roy S.C. (2019). High photoelectrochemical activity of CuO nanoflakes grown on cu foil. Electrochim. Acta.

[67] Iqbal M., Thebo A.A., Shah A.H., Iqbal A., Thebo K.H., Phulpoto S., Mohsin M.A. (2017). Influence of mn-doping on the photocatalytic and solar cell efficiency of CuO nanowires. Inorg. Chem. Commun..

[68] Mobini S., Meshkani F., Rezaei M. (2017). Synthesis and characterization of nanocrystalline copper–chromium catalyst and its application in the oxidation of carbon monoxide. Process Saf. Environ. Prot..

[69] Rahimi M.G., Wang A., Ma G., Han N., Chen Y. (2020). A one-pot synthesis of a monolithic Cu_2_O/Cu catalyst for efficient ozone decomposition. RSC Adv..

[70] Zhang P., Wang R., He M., Lang J., Xu S., Yan X. (2016). 3D hierarchical co/CoO-graphene-carbonized melamine foam as a superior cathode toward long-life lithium oxygen batteries. Adv. Funct. Mater..

[71] Dai K., Zhang N., Zhang L., Yin L., Zhao Y., Zhang B. (2021). Self-supported co/CoO anchored on N-doped carbon composite as bifunctional electrocatalyst for efficient overall water splitting. Chem. Eng. J..

[72] Wei Y., Ma Z., Liu B., Yang J., Wu D., Zhang Y., Zhang Y., Xu C.C., Nie R. (2024). Phase transition induced hydrogen activation for enhanced furfural reductive amination over a CoCu bimetallic catalyst. Chem. Sci..

[73] Zhang C., Chen J., Chen W., Liu J., Chen D. (2023). Hydrothermal synthesis of Cu_2_O/CuO/hierarchical porous N-doped activated carbon with exceptional electrochemical performance. J. Energy Storage.

[74] Bai J., Yang L., Dai B., Ding Y., Wang Q., Han J., Zhu J. (2018). Synthesis of CuO-Cu_2_O@graphene nanosheet arrays with accurate hybrid nanostructures and tunable electrochemical properties. Appl. Surf. Sci..

[75] Ye J.J., Li P.H., Zhang H.R., Song Z.Y., Fan T., Zhang W., Tian J., Huang T., Qian Y., Hou Z. (2023). Manipulating Oxygen Vacancies to Spur Ion Kinetics in V_2_ O_5_ Structures for Superior Aqueous Zinc-Ion Batteries. Adv. Funct. Mater..

[76] Yang Y., Lau K.Y., Zheng J., Dong J., Wang L., Wang W., Xu B., Qiu J., Liu X. (2023). Coupled Femtosecond Laser Assisted Doping and Fragmentation of MoO_3_ Nanosheets Generates Plasmonic QDs with Strong NLO Response. Adv. Opt. Mater..

[77] Lin W., Yu Y., Fang Y., Liu J., Li X., Wang J., Zhang Y., Wang C., Wang L., Yu X. (2021). Oxygen Vacancy-Enhanced Photoelectrochemical Water Splitting of WO_3_/NiFe-Layered Double Hydroxide Photoanodes. Langmuir.

[78] Wang Z., Lin R., Huo Y., Li H., Wang L. (2022). Formation, Detection, and Function of Oxygen Vacancy in Metal Oxides for Solar Energy Conversion. Adv. Funct. Mater..

[79] Fang Z., Rehman S.U., Sun M., Yuan Y., Jin S., Bi H. (2018). Hybrid NiO–CuO mesoporous nanowire array with abundant oxygen vacancies and a hollow structure as a high-performance asymmetric supercapacitor. J. Mater. Chem. A.

[80] Wang W., Zhang M., Mi R., Liu Y., Chen J. (2021). Facile synthesis of porous Co_3_O_4_ nanosheets containing abundant oxygen vacancies for boosted lithium-ion storage. J. Alloys Compd..

[81] Zhu X., Zhu P., Li Y., Liu Y. (2024). Nickel-cobalt oxide nanowires with oxygen vacancies supported on CVD graphene networks for all-solid-state asymmetric supercapacitors. J. Energy Storage.

[82] Abdul Sammed K., Kumar A., Farid A., Zhang W., Rehman Akbar A., Ali M., Ajmal S., Yasin G., Ullah N., Pan L. (2024). Exploration of the role of oxygen-deficiencies coupled with Ni-doped V_2_O_5_ nanosheets anchored on carbon nanocoils for high-performance supercapacitor device. Chem. Eng. J..

[83] Wu Z.-S., Parvez K., Feng X., Müllen K. (2014). Photolithographic fabrication of high-performance all-solid-state graphene-based planar micro-supercapacitors with different interdigital fingers. J. Mater. Chem. A.

[84] Sundriyal P., Bhattacharya S. (2019). Scalable Micro-fabrication of Flexible, Solid-State, Inexpensive, and High-Performance Planar Micro-supercapacitors through Inkjet Printing. ACS Appl. Energy Mater..

[85] Jiang J.-Z., Gu Y.-J., Wen W., Ye Z.-Z., Wu J.-M. (2024). Copper-doped ceria on carbon fibers for high specific capacitance supercapacitors. J. Energy Storage.

[86] Kumar S.K., Reddy L., Hatshan M.R., Roy N., Kim J.S., Joo S.W. (2024). Comprehensive characterization of octahedral Co_3_O_4_ doped with Cu induces oxygen vacancies as a battery-type redox active electrode material for supercapacitors. Ceram. Int..

[87] Chen Y., Li X., Bi Z., Li G., He X., Gao X. (2018). Stamp-assisted printing of nanotextured electrodes for high-performance flexible planar micro-supercapacitors. Chem. Eng. J..

[88] Zhang P., Zhu F., Wang F., Wang J., Dong R., Zhuang X., Schmidt O.G., Feng X. (2017). Stimulus-Responsive Micro-Supercapacitors with Ultrahigh Energy Density and Reversible Electrochromic Window. Adv. Mater..

[89] Li J., Guo S., Chen K., Zhao M., Wu W., Xia X., Zhao J. (2024). CuO nanoparticles embedded in laser-induced graphene for flexible planar micro-supercapacitors. Surf. Interfaces.

[90] Lu Y., Jiang K., Chen D., Shen G. (2019). Wearable sweat monitoring system with integrated micro-supercapacitors. Nano Energy.

