# Supplementary material for: A Computer Numerical Control Wire Electrical Discharge Machining Strategy for Fabricating Cobalt–Copper Bimetallic Oxide Maze-like Micro-Supercapacitors
Source: Micromachines (Basel). 2026 Apr 23;17(5):516. doi: 10.3390/mi17050516 (PMC13208403; doi:10.3390/mi17050516)
Supplement: Supplementary file 1 [file micromachines-17-00516-s001.zip › micromachines-4263420-supplementary.pdf]

Supplementary

A computer numerical control wire electrical discharge machining strategy for fabricating cobalt-copper bimetallic oxide maze-like micro-supercapacitors

Ziliang Chen, Rui Xie, Chunlong Chen, Yiwei Zheng, Jianping Deng, Dawei Liu, Binbin Zheng, Wenxia Wang, Igor Zhitomirsky and Ri Chen

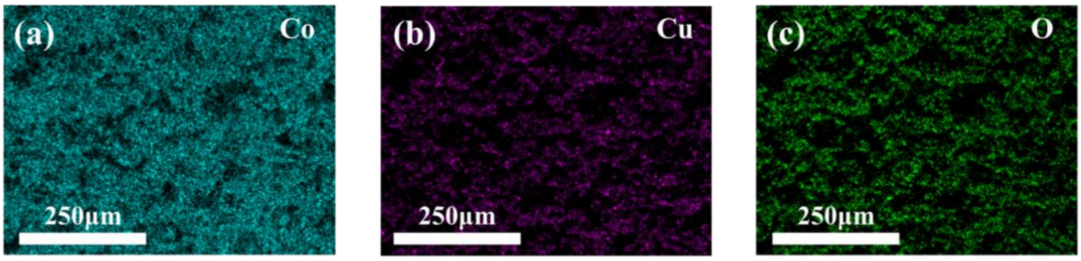

Figure S1. EDS mapping of individual elements for the $CoCuO_x$ electrode:(a) Co, (b) Cu, and (c) O.

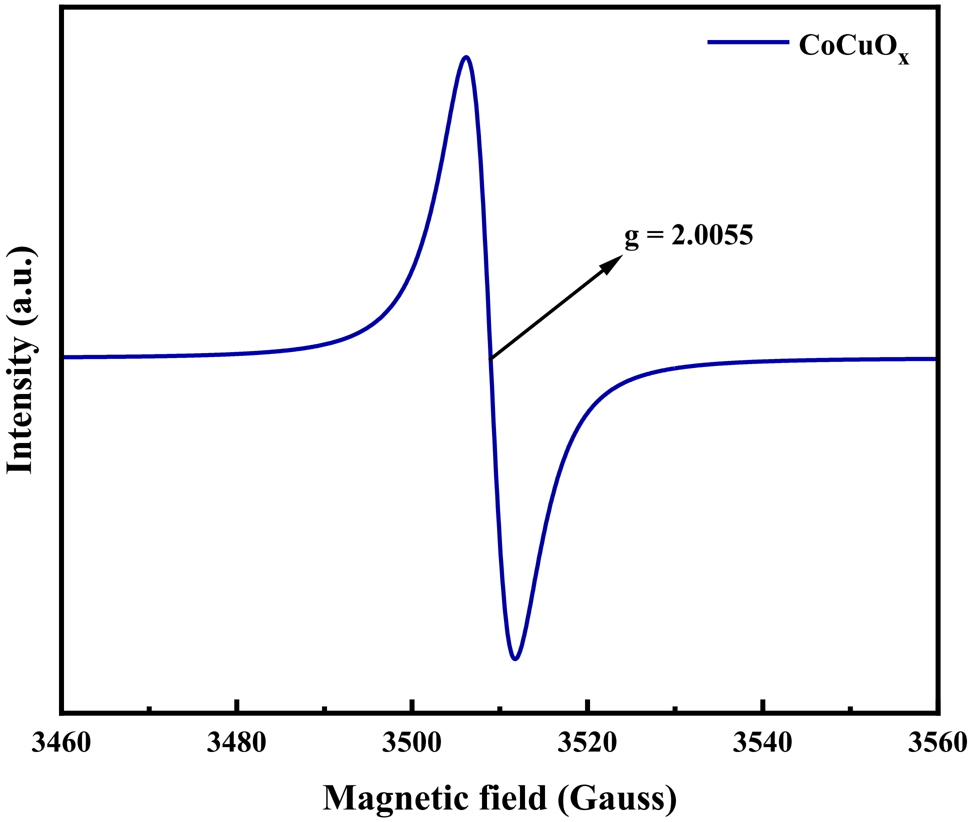

Figure S2. EPR spectrum of the $CoCuO_x$ electrode.

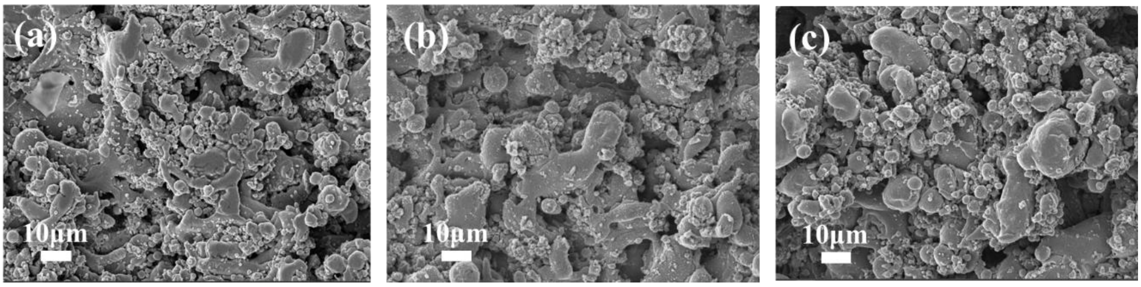

Figure S3. SEM (scale bar: 10 μm) of CNCWEDM processed at different machining voltages: (a) 60 V, (b) 80 V, and (c) 100 V.

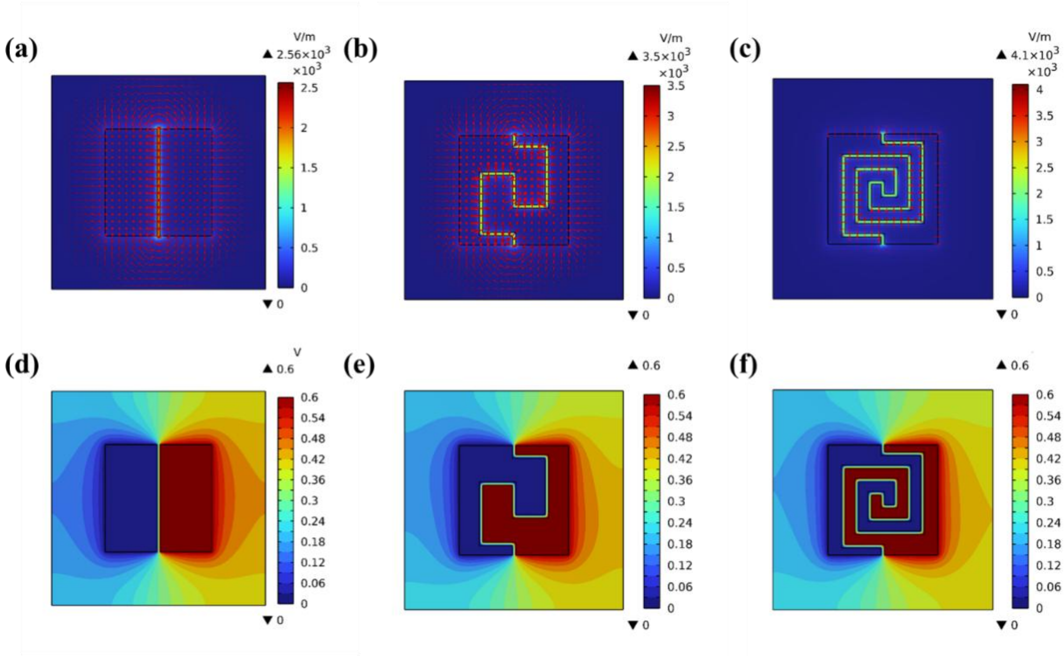

Figure S4. Electrostatic field and potential distributions at 0.6 V for different electrode structures. (a–c) Electric field intensity distributions for electrode structures 1-CoCuMMSCs60, 2-CoCuMMSCs60, and 3-CoCuMMSCs60, respectively. (d–f) Potential distribution maps for the corresponding structures.

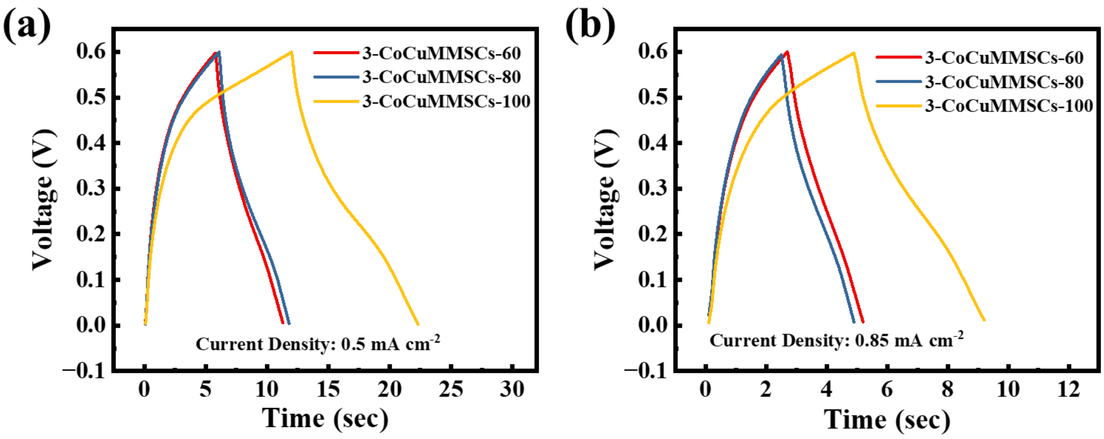

Figure S5. GCD for CoCuMMSCs at (a) 0.5 mA cm^{-2} and (b) 0.85 mA cm^{-2}.
